# Methionine mitigates aflatoxicosis in quail chicks by improving gut microbiota, immunity, and meat quality

**DOI:** 10.1016/j.toxrep.2024.101875

**Published:** 2024-12-18

**Authors:** Adel Ghorbani, Mahmoud Ghazaghi, Farzad Bagherzadeh-Kasmani, Mohammad Rokouei, Mehran Mehri

**Affiliations:** Department of Animal Sciences, Faculty of Agriculture, University of Zabol, Sistan 98661-5538, Iran

**Keywords:** Aflatoxicosis, Antioxidant, Food quality, Immunology, Malondialdehyde, Quail

## Abstract

This study aimed to investigate the effects of dietary methionine (Met) supplementation on performance, immunity, and meat quality in growing Japanese quail exposed to aflatoxin B_1_ (AFB_1_)-contaminated diets. Nine experimental diets were formulated, incorporating three levels of dietary Met (5.0, 6.0, and 7.0 g/kg) and three levels of AFB_1_ (0.0, 2.5, and 5.0 mg/kg) in a completely randomized design and fed from d 8 post-hatch to d 35 of age. The results revealed that increasing dietary Met levels significantly improved body weight gain (BWG; P < 0.001), feed conversion ratio (FCR; P < 0.001), and feed intake (FI; P < 0.001), while counteracting the negative effects of AFB_1_ on these performance parameters. Dietary Met supplementation also exerted a protective effect against elevated hepatic enzyme levels (AST, P < 0.001; ALT, P < 0.001; ALP, P = 0.001; and LDH, P < 0.001) and serum uric acid levels (P < 0.001) induced by AFB_1_. Furthermore, dietary Met enhanced humoral immunity responses by increasing antibody production against sheep red blood cell antigen (P < 0.001) and hemagglutination inhibition response (P < 0.001), mitigating the AFB_1_-induced immune impairment. Meat quality parameters, including pH (P = 0.04), drip loss (P < 0.00_1_), and malondialdehyde concentration (P < 0.001), were significantly influenced by the interaction between dietary Met and AFB_1_. Lastly, dietary Met supplementation effectively counteracted AFB_1_'s detrimental effects on ileal lactic acid bacteria populations (P < 0.001). In conclusion, dietary Met supplementation shows promise as a nutritional intervention to alleviate the harmful effects of AFB_1_ exposure in Japanese quail, particularly in improving food quality and overall health.

## Graphical Abstract

**Figure:**
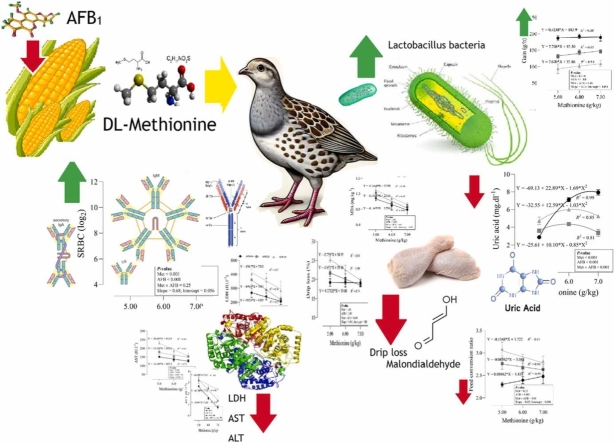

## 1 Introduction

Aflatoxin B_1_ (AFB_1_) is a toxic fungal metabolite produced by *Aspergillus* species, which frequently contaminates agricultural commodities, posing significant health risks to both humans and animals [1]. In poultry, AFB_1_ impairs growth performance and increases reactive oxygen species (ROS) production [2], contributing to oxidative stress that negatively impacts the animals' overall health [3]. The deleterious effects of AFB_1_ on poultry have prompted the investigation of various strategies to counteract aflatoxicosis, particularly in relation to its impact on growth, immunity, and meat quality. One promising approach involves the use of dietary supplements, such as specific amino acids, to mitigate the toxic effects of AFB_1_. Methionine (Met), an essential amino acid, has shown potential in alleviating aflatoxicosis due to its capacity to stimulate the metabolism and excretion of AFB_1_ in birds [4], [5]. Research has demonstrated that Met supplementation can enhance the activity of certain hepatic metabolites, including glutathione and acetylcholinesterase, which play crucial roles in mycotoxin detoxification [5]. Moreover, Met has been found to exert protective effects on the liver by reducing oxidative stress [6], ultimately improving liver function [7].

Despite the promising benefits of Met supplementation, the optimal dose and duration of administration in aflatoxicosis remain unclear, as they may depend on factors such as the poultry species, age, sex, health status, and mycotoxin contamination levels. Consequently, there is a need to investigate the influence of Met supplementation on various aspects of poultry health and performance under different AFB_1_ exposure scenarios.

This study aims to evaluate the effects of varying AFB_1_ dosages and excess dietary Met on growth performance, blood parameters, immunity, meat quality, and intestinal microbiota composition in Japanese quail chicks during their growth phase. By exploring the potential protective effects of Met supplementation, we can contribute to the development of more effective strategies for mitigating the detrimental effects of AFB_1_ in poultry and enhancing overall health, productivity, and food safety.

## 2 Materials and methods

### 2.1 Ethics statement

This experimental protocol has been approved by both the animal ethics committee at the University of Zabol and the Iranian Council of Animal Care. The study adhered to the ARRIVE guidelines and the NIH Guidelines for Animal Care and Use [8].

### 2.2 Bird management

One-day-old Japanese quail chicks (*Coturnix coturnix Japonica*) were obtained from the Research Center of the Research Institute of Zabol, Iran. The chicks were fed a grower diet based on NRC (1994) recommendations from hatch to 7 days of age. At d 8, a total of 675 quail chicks were randomly allotted to 45-floor pens comprising 9 treatments with 5 replicate pens with 15 birds per pen. Body weight and feed intake were recorded weekly to measure body weight gain (BWG) and feed conversion ratio (FCR). The temperature was held at 26ºC ± 2.50 in the third week of age afterward, with a relative humidity of 60 % ± 3.30. The lighting program was 16 L:8D during the study.

### 2.3 Experimental diets

Nine experimental diets (Table 1) were formulated to include three levels of dietary Met (5.0, 6.0, and 7.0 g/kg) and three levels of AFB_1_ (0.0, 2.5, and 5.0 mg/kg). Each experimental diet was fed to one of the nine groups of birds from 8 to 35 d of age, providing 3 × 3 factorial arrangements of studied nutrients (Table 2). Prior to beginning the experiment, all protein-containing ingredients in the basal diet were analyzed for crude protein (CP) and amino acid profiles [9], [10]. These analyzed values were then used to formulate the diet. After mixing the feed ingredients, amino acid content of the basal diet was assessed. Sample preparation involved hydrolyzing the samples in 6 N hydrochloric acid at 110°C for 24 h under nitrogen. To measure Met and cysteine (Cys), performic acid oxidation preceded acid hydrolysis, while tryptophan (Trp) samples were hydrolyzed using barium hydroxide [11]. Amino acid separation was conducted using a Waters HPLC system, which included a 1525 Binary HPLC pump, a 2487 Dual λ absorbance detector at 254 nm, Breeze software, and a Rheodyne 7725 injection valve with a 20 μl sample loop. A Pico Tag column (3.9 × 150 mm, particle size 5 μm) was used for chromatographic separation.

**Table 1:** Composition of the experimental diets.

Ingredient	Aflatoxin B_1_ (g/kg)								
	0.0			2.5			5.0		
	Dietary methionine (g/kg)								
	5.0	6.0	7.0	5.0	6.0	7.0	5.0	6.0	7.0
Corn	39.10	39.23	39.36	43.68	42.98	45.16	37.36	37.36	37.36
Soybean meal	27.26	27.35	27.45	26.97	27.11	26.47	27.69	27.69	27.69
Corn gluten Meal	17.90	17.62	17.32	17.56	17.35	16.36	18.02	18.02	18.02
Rice	6.29	6.24	6.19	-	-	-	-	-	-
Rice-AFB_1_ (64.21 ppm)	-	-	-	3.89	3.89	3.89	7.79	7.79	7.79
Sunflower oil	2.21	2.21	2.21	0.68	0.68	0.68	2.21	2.21	2.21
Corn starch	3.00	3.00	3.00	3.00	3.00	3.00	3.00	2.89	2.79
Limestone	1.48	1.48	1.48	1.49	1.48	1.48	1.47	1.47	1.47
DCP	0.71	0.71	0.71	0.70	0.70	0.71	0.71	0.71	0.71
DL-Methionine	0.08	0.18	0.29	0.07	0.18	0.29	0.08	0.18	0.28
Sodium bicarbonate	0.79	0.79	0.79	0.78	0.79	0.79	0.80	0.80	0.80
Mineral premix^1^	0.25	0.25	0.25	0.25	0.25	0.25	0.25	0.25	0.25
Vitamin premix^2^	0.25	0.25	0.25	0.25	0.25	0.25	0.25	0.25	0.25
L-Lysine.HCl	0.50	0.50	0.50	0.51	0.50	0.49	0.21	0.21	0.21
NaCl	0.06	0.06	0.06	0.04	0.04	0.04	0.04	0.04	0.04
L-Threonine	0.13	0.13	0.13	0.13	0.13	0.13	0.13	0.13	0.13

^1^Mineral premix provided per kilogram of diet: Mn (from MnSO4·H2O), 65 mg; Zn (from ZnO), 55 mg; Fe (from FeSO4·7H2O), 50 mg; Cu (from CuSO4·5H2O), 8 mg; I [from Ca (IO_3_)2·H_2_O], 1.8 mg; Se, 0.30 mg; Co (from Co_2_O_3_), 0.20 mg; Mo, 0.16 mg.

^2^Vitamin premix provided per kilogram of diet: vitamin A (from vitamin A acetate), 11,500 U; cholecalciferol, 2100 U; vitamin E (from dl-α-tocopheryl acetate), 22 U; vitamin B_12_, 0.60 mg; riboflavin, 4.4 mg; nicotinamide, 40 mg; calcium pantothenate, 35 mg; menadione (from menadione dimethyl-pyrimidinol), 1.50 mg; folic acid, 0.80 mg; thiamine, 3 mg; pyridoxine, 10 mg; biotin, 1 mg; choline chloride, 560 mg; ethoxyquin, 125 mg.

^3^DEB: dietary electrolyte balance represents dietary Na + K – Cl in mEq/kg of diet.

**Table 2:** Nutrient composition of the experimental diets.

Nutrients	Aflatoxin B_1_ (g/kg)								
	0.0			2.5			5.0		
	Dietary methionine (g/kg)								
	5.0	6.0	7.0	5.0	6.0	7.0	5.0	6.0	7.0
ME (kcal/kg)	2950	2950	2950	2950	2950	2950	2900	2900	2900
CP (g/kg)	260	260	260	260	260	260	260	261	262
Lysine (g/kg)	13.0	13.0	13.0	13.0	13.0	13.0	13.0	13.0	13.0
Threonine (g/kg)	9.10	9.10	9.10	9.10	9.10	9.10	9.10	9.10	9.10
Tryptophan (g/kg)	2.10	2.10	2.10	2.10	2.10	2.10	2.10	2.10	2.10
Methionine (g/kg)	5.00	6.00	7.00	5.00	6.00	7.00	5.00	6.00	7.00
Methionine + Cysteine (g/kg)	8.40	8.40	8.40	8.40	8.40	8.40	8.40	9.40	10.40
Ca (g/kg)	8.00	8.00	8.00	8.00	8.00	8.00	8.00	8.00	8.00
P _available_ (g/kg)	3.00	3.00	3.00	3.00	3.00	3.00	3.00	3.00	3.00
DEB (mEq/kg)^3^	250	250	250	250	250	250	250	250	250
AFB_1_ (ppm)	0.0	0.0	0.0	2.50	2.50	2.50	5.00	5.00	5.00

### 2.4 Preparation of AFB_1_

The PTCC-5286 strain of *Aspergillus parasiticus*, a fungus known to produce AFB_1_, was grown on corn grain under controlled temperature and stirring, following a commonly used method for AFB_1_ synthesis [12]. The concentration of AFB_1_ in the sample was determined at 64.21 mg/kg, using high-performance liquid chromatography (HPLC) [13]. Contaminated corn was added to the experimental diets to achieve the desired AFB_1_ levels according to the experimental design.

### 2.5 Serum biochemical analysis

On day 35, blood samples were collected from two birds per replicate via jugular vein puncture, and serum was separated. Serum parameters, including lactate dehydrogenase (LDH), alkaline phosphatase (ALP), total protein (TP), aspartate aminotransferase (AST), alanine aminotransferase (ALT), glucose, triglyceride (TG), and uric acid (UA) were analyzed using a spectrophotometer (UNIKON 933; Kontron Co. Ltd., Milan, Italy) and commercially available kits (Parsazmun, Tehran, Iran). The procedures were conducted according to the manufacturer's recommendations.

### 2.6 Humoral immunity responses

Four birds in each replicate were challenged with sheep red blood cell (SRBC) antigens at d 18 and 25, and after the second challenge, antibody production against SRBC antigen was measured by hemagglutination inhibition test in the serum samples according to Cheema et al. [14]. Four birds in each pen were vaccinated with the Newcastle virus (NDV-B1 strain) vaccine through intraocular administration on d 14 and 21. At d 35, blood was collected from 20 birds per experimental group through the jugular vein, and then serum was separated from the blood clot. The hemagglutination inhibition (HI) method was used to determine the titer of antibodies produced against the Newcastle virus [15].

### 2.7 Malondialdehyde measurement

At the end of experiment, four birds from each replicate were sacrificed by CO_2_ asphyxiation, and their deboned meat was stored at −20°C for 30 days. As previously described by Botsoglou et al. [16], one gram of grounded meat sample was weighed and homogenized (Polytron homogenizer, PCU, Switzerland) with 4 mL of 5 % aqueous trichloroacetic acid (TCA) and 2.5 mL of 0.8 % butylated hydroxytoluene, and then centrifuged at 3000 × g for 3 min. The top hexane layer was discarded, and the bottom layer was filtered and made to a 5 mL volume with 5 % TCA and then added to a screw-capped tube containing 3 mL of 0.8 % aqueous 2-thiobarbituric acid (TBA). Finally, tubes were placed in a 70°C water bath for 30 min, then immediately cooled under tap water and submitted to spectrophotometry (UNIKON 933, Kontron Co. Ltd., Milan, Italy). The height of the third-order derivative peak that appeared at 521.5 nm was used for the calculation of the malondialdehyde (MDA) concentration in the samples. Tetraethoxypropane (1, 1, 3, 3- Tetraethoxy propane, T9889, 97 %, Sigma, USA.) was the MDA precursor in the standard curve. The concentration of MDA was expressed as milligrams per kilogram of meat sample.

### 2.8 Bacterial populations of ileal content

The ileal contents of 4 birds in each replicate were separately collected into the sterile tubes for serial dilution, as described by Ghazaghi et al., 2014. In brief, one g of ileal digesta was added into the test tube containing 9 mL of sterilized phosphate-buffered saline (PBS; pH = 7.4, 0.1 M). Microbial populations were determined by serial dilution (10−4 to 10−6) of ileal samples before inoculation onto Petri dishes. Plates for lactic acid bacteria (LAB; grown in deMan, Rogosa and Sharpe, MRS agar) colonies were counted between 24 and 48 h after inoculation. For E-coli counting, tenfold dilutions were prepared, and 0.1 mL of each dilution was spread over the surface of MacConkey agar plates and incubated at 37ᵒC for 24 h. Colony-forming units were defined as distinct colonies measuring at least one mm in diameter.

### 2.9 Statistical analysis

Statistical analyses were performed with GraphPad Prism (GraphPad Prism Software Inc., San Diego, CA). To assess the effect of dietary Met at various concentrations of AFB_1_, a two-way ANOVA test was performed, followed by subsequent analyses using linear or quadratic regression models. Levels of significance were set at *P* < 0.05.

## 3 Results

### 3.1 Growth performance

**Fig. 1:**
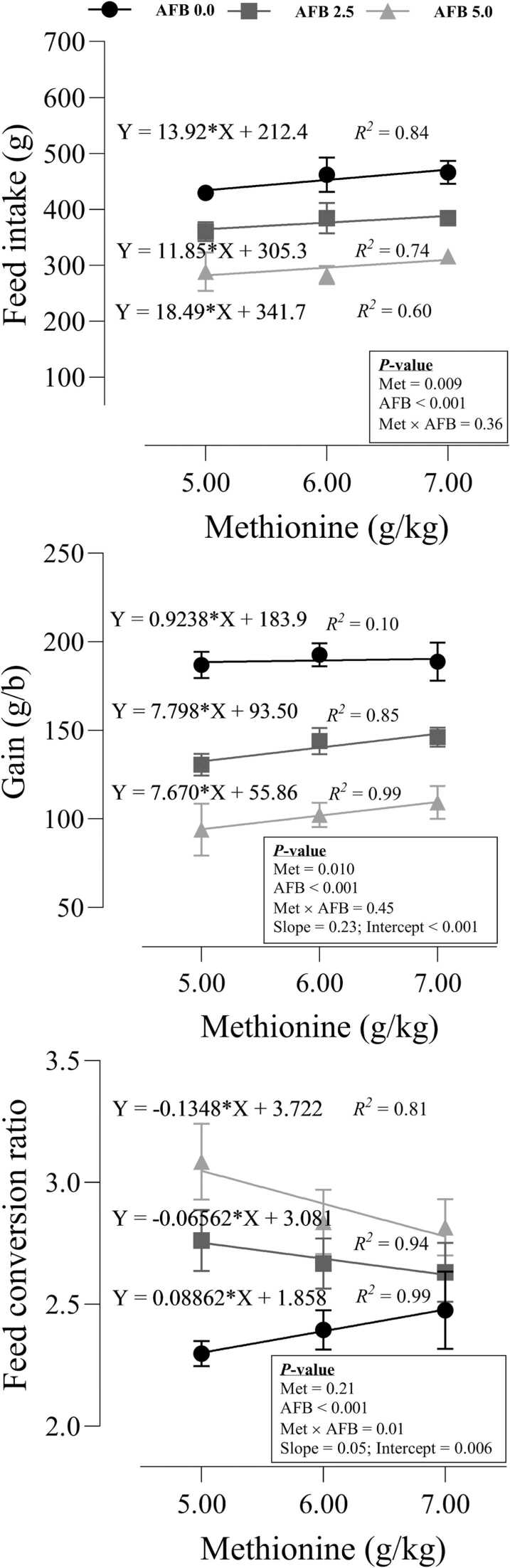
Growth responses of quail chicks fed different levels of dietary methionine when exposed to aflatoxin B_1_. The intercept and slope of the fitted lines were evaluated statistically and probabilities of these parameters were provided.

The effects of dietary Met and AFB_1_ on growth performance for Japanese quail were shown in Fig. 1. Increasing dietary Met linearly increased FI (P = 0.009) independent of AFB_1_, while including AFB_1_ decreased FI (P < 0.001). There was no interaction between Met and AFB_1_ (P = 0.36). The significant intercept (P < 0.001) of the linear model proved the negative effects of AFB_1_ on FI of quail chicks, while the non-significant slope (P = 0.87) revealed that increasing dietary Met increased the FI with the same progression between non-toxic and AFB_1_-contaminated groups. The same pattern was seen for BWG except for the non-toxic group, where increasing dietary Met did not increase the BWG of quail chicks. The non-significant slope (P = 0.23) of the linear models revealed that the increasing effect of Met on BWG was the same at two levels of AFB_1_. Dietary Met and AFB_1_ interacted on FCR (P = 0.01), where increasing dietary Met in the toxic-free group adversely increased FCR, but higher dietary Met in the birds fed AFB_1_ decreased FCR. The significant intercept (P = 0.006) of the model indicated that FCR was lower in toxic-free birds than those fed AFB_1_ at all dietary levels of Met. The significant slope of the linear model revealed that the decreasing effect of Met on FCR in birds fed AFB_1_ at the highest level of AFB_1_ (5.0 g/kg) was higher (b = −0.135) than that (b = −0.066) in birds fed lower level of AFB_1_ (2.5 g/kg).

### 3.2 Hepatic enzymes

**Fig. 2:**
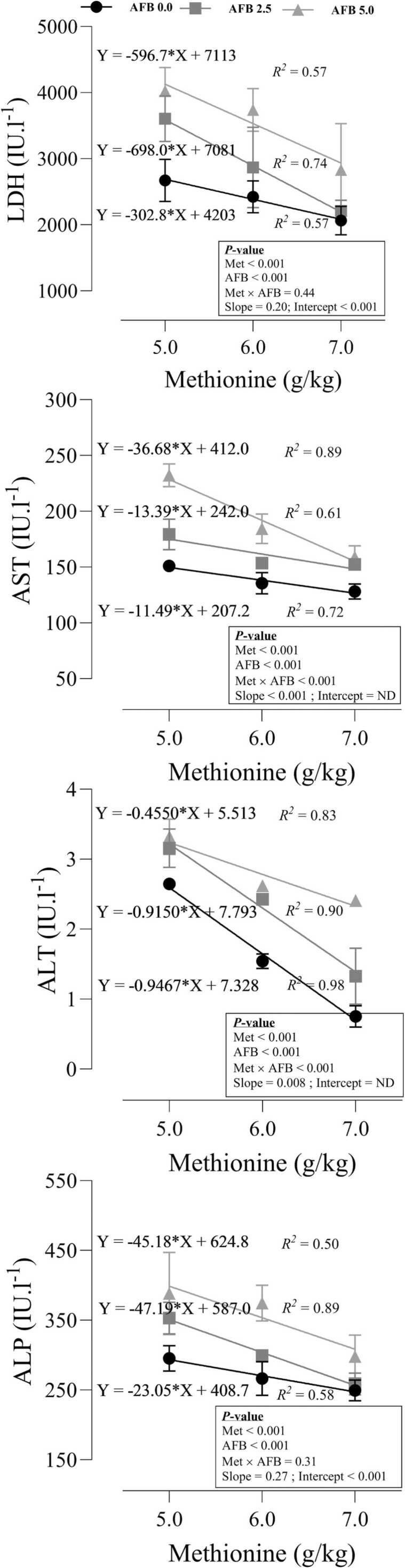
Response of hepatic enzymes in quail chicks fed different levels of dietary methionine when exposed to aflatoxin B_1_. The intercept and slope of the fitted lines were evaluated statistically and probabilities of these parameters were provided.

The response of hepatic enzymes to dietary Met and AFB_1_ was shown in Fig. 2. The individual effects of Met and AFB_1_ on the LDH level were significant, where the increasing dietary Met decreased (P < 0.001) LDH level in the blood, but AFB_1_ increased the hepatic concentration of LDH in the blood (P < 0.001). No interaction was seen between Met and AFB_1_ on this response (P = 0.44). The significant intercept (P < 0.001) of the model exhibited that despite dietary Met, including AFB_1_ in the diet increased the blood concentration of LDH. In addition, the non-significant slope (P = 0.20) of the linear models indicated that increasing levels of Met led to a consistent decrease in the LDH concentration with no significant difference, despite AFB_1_ in the diet. The significant interaction effect of Met × AFB_1_ (P < 0.001) and the significant effect of the slope (P < 0.001) was seen in the concentration of AST in blood. Depending on the concentration of AFB_1_ in diet, increasing dietary Met decreased the concentration of AST with different power, where increasing dietary Met in birds fed the highest AFB_1_ level decreased AST with the steepest slope (b = −36.68), followed by 2.5 g/kg AFB_1_ (b = −13.39) and toxic-free (b = −11.49) groups. The same pattern was observed for ALT, where the interaction between Met × AFB_1_ (P < 0.001) and the significant effect of the slope (P = 0.008) was detected. In contrast to AST, increasing dietary Met in birds fed a toxic-free diet decreased ALT with the steepest slope (b = −0.947), followed by 2.5 g/kg AFB_1_ (b = −0.915) and 5.0 g/kg AFB_1_ (b = −0.455) groups. In terms of ALP, increasing dietary Met decreased ALP in all groups with the same slope (P = 0.27), but the significant intercept (P < 0.001) revealed that the concentration of ALP in the toxic-free group was lowest at three levels of dietary Met, followed by 2.5, and 5.0 g/kg AFB_1_ groups.

### 3.3 Blood biochemical profile

**Fig. 3:**
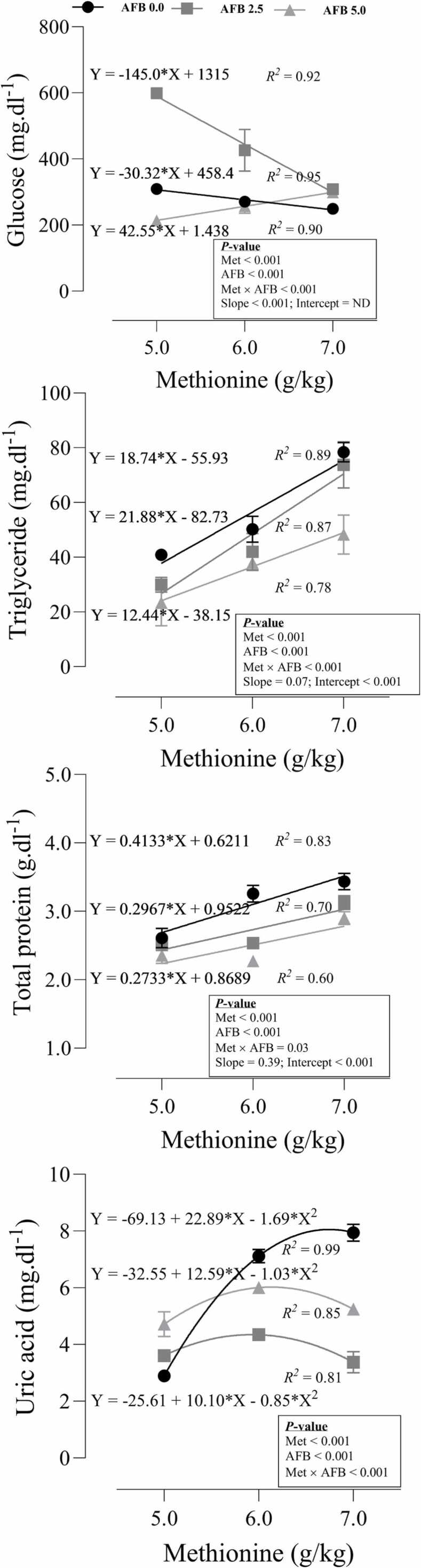
Blood biochemical profile of quail chicks fed different levels of dietary methionine when exposed to aflatoxin B_1_. The intercept and slope of the fitted lines were evaluated statistically and probabilities of these parameters were provided.

The response of some biochemical variables to dietary Met and AFB_1_ was shown in Fig. 3. The interaction of Met and AFB_1_ was significant (P < 0.001) on the glucose concentration in the blood. In the 5.0 g/kg AFB_1_ group, increasing dietary Met increased the concentration of blood glucose while decreased the glucose level in other groups. The significant slope (P < 0.001) of the linear model showed that the decrease in blood glucose was greater for birds exposed to 2.5 g/kg AFB_1_ by up to 80 % than for those fed toxin-free diet. There is a noticeable phenomenon within the plasma levels of glucose, in which the highest level of dietary Met intake (7.0 g/kg of diet) leads to a close level of glucose across all groups. Hence, the level of plasma glucose, despite their exposure to AFB_1_, tends to stabilize at a similar level once the concentration of dietary Met is sufficiently high. Similarly, the interaction between Met and AFB_1_ was significant (P < 0.001) on the serum concentration of TG. A significant intercept (P < 0.001) was observed within the model, suggesting that AFB_1_ resulted in a lower concentration of TG, and the linear increase in dietary Met led to an increase in the level of TG in the bloodstream. However, the slope of the model tended to be significant (P = 0.07). The interaction between dietary Met and AFB_1_ (P = 0.03) was seen in the blood concentration of TP. The significant intercept of the model (P < 0.001) revealed that AFB_1_ caused a decrease in the level of TP, while the increasing dietary Met reversed it. A significant quadratic term in the model of blood UA levels (P < 0.001) indicates that the response to dietary Met was nonlinear. In the groups exposed to AFB_1_, the concentration of UA had its lowest point at the highest level of dietary Met. Conversely, in the toxic-free group, the linear section of the model indicated a more significant effect of increasing dietary Met, leading to higher UA levels in the bloodstream.

### 3.4 Humoral immunity

**Fig. 4:**
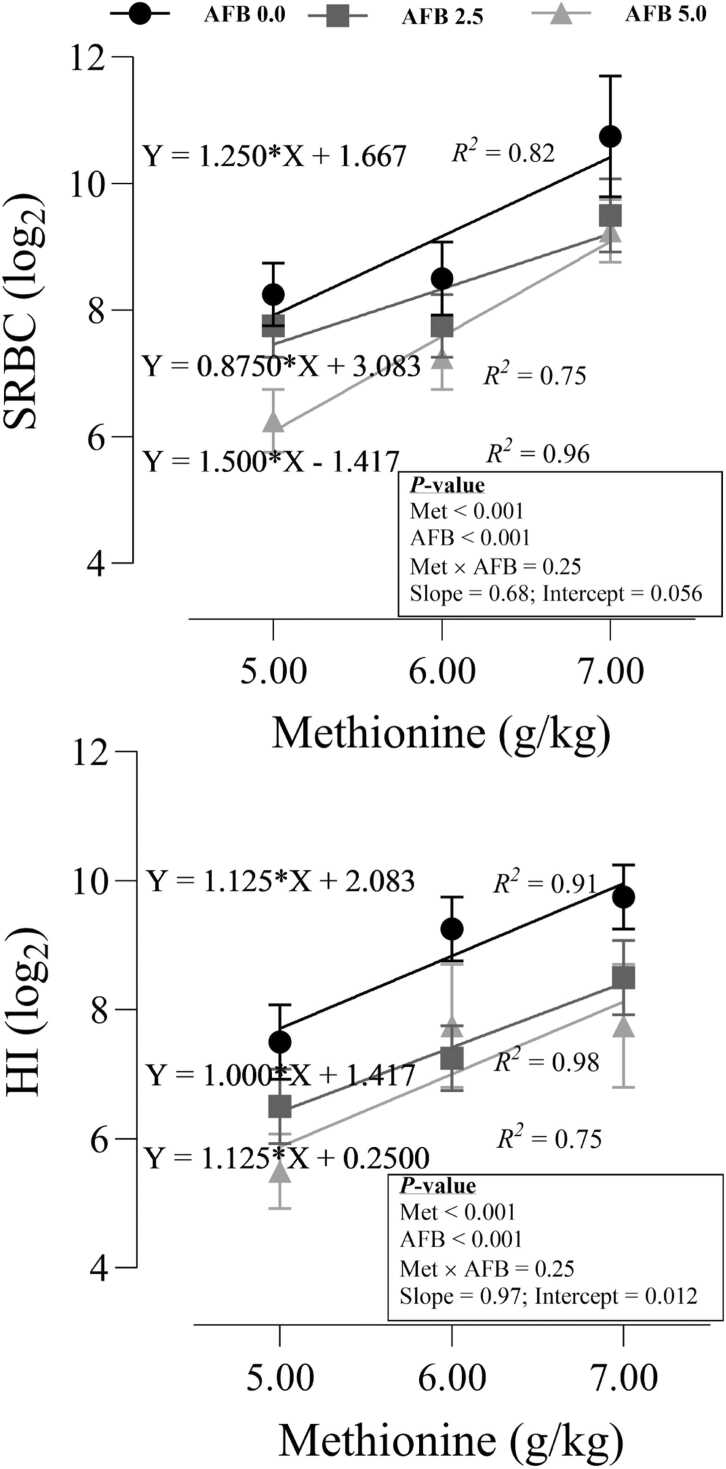
Humoral responses (sheep red blood cell, SRBC; hemagglutination inhibition, HI) in quail chicks fed different levels of dietary methionine when exposed to aflatoxin B_1_. The intercept and slope of the fitted lines were evaluated statistically, and probabilities of these parameters were provided.

As shown in Fig. 4, AFB_1_ decreased (P < 0.001) antibody production against SRBC antigen, while increasing dietary Met linearly increased (P < 0.001) SRBC response in all experimental groups. A similar pattern was seen for the HI test, where the significant reduction in HI response in birds fed AFB_1_ (P < 0.001) was reversed by increasing dietary Met (P < 0.001). The intercept of models for SRBC (P = 0.056) and HI (P = 0.012) revealed that including AFB_1_ in diet had a profound effect on immunity responses, however, the slope of the models was not significant, and increasing dietary Met increased the immunity responses with the same progression.

### 3.5 Meat quality

**Fig. 5:**
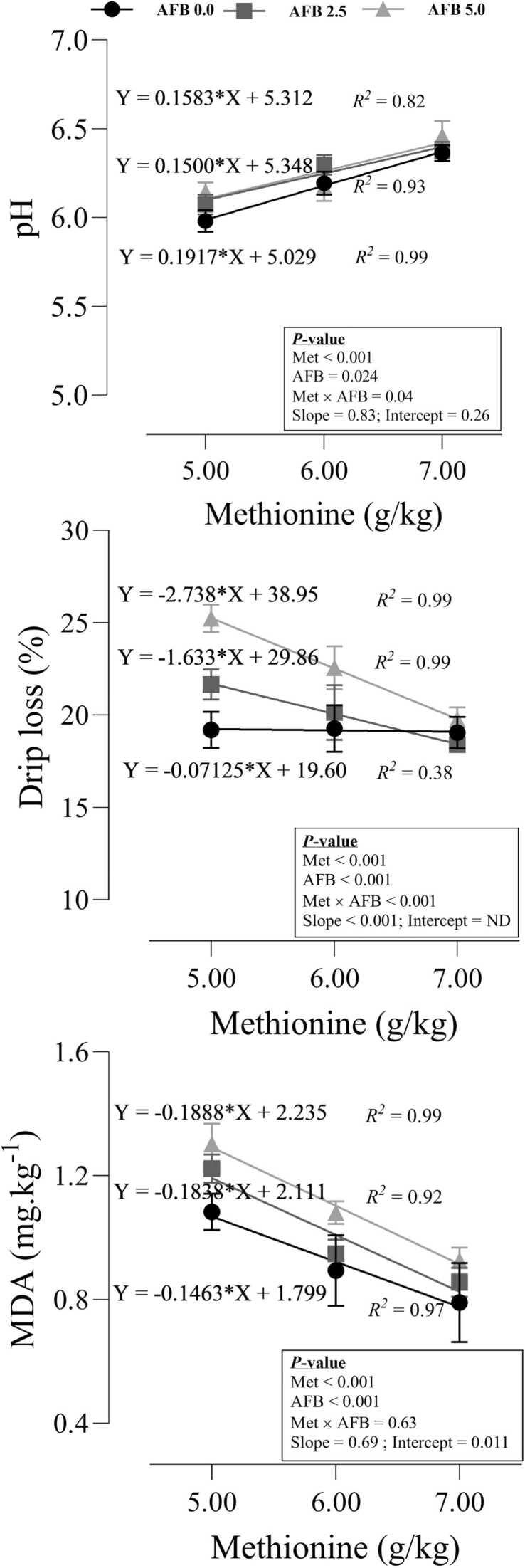
Drip loss and malondialdehyde (MDA) concentration and pH in meat samples of quail chicks fed different levels of dietary methionine when exposed to aflatoxin B_1_. The intercept and slope of the fitted lines were evaluated statistically and probabilities of these parameters were provided.

The responses of meat quality attributes were shown in Fig. 5. There was an interaction between Met and AFB_1_ (P = 0.04) on the pH of meat samples and a potential synergism was observed between the experimental inputs. No significant intercept (P = 0.26) and slope (P = 0.83) of the models revealed that the increment in pH was identical across the treatments. A high interaction between Met and AFB_1_ (P < 0.001) was seen on drip loss, where the increase in drip loss caused by AFB_1_ was reversed by increasing dietary Met. The significant slope of the linear models (P < 0.001) showed that, although the impact of dietary Met was negligible on the drip loss in the toxic-free group, increasing dietary Met significantly decreased the drip loss in the meat samples of the birds fed AFB_1_. AFB_1_ (P < 0.001) increased the production of MDA in meat samples, while dietary Met (P < 0.001) decreased the MDA without significant interaction (P = 0.63). Despite the difference in intercept (P = 0.011) between the models, the lack of significance (P = 0.69) in the slope of the models indicates that the reduction in MDA by increasing dietary Met follows a similar pattern.

### 3.6 Intestinal microbiota communities

**Fig. 6:**
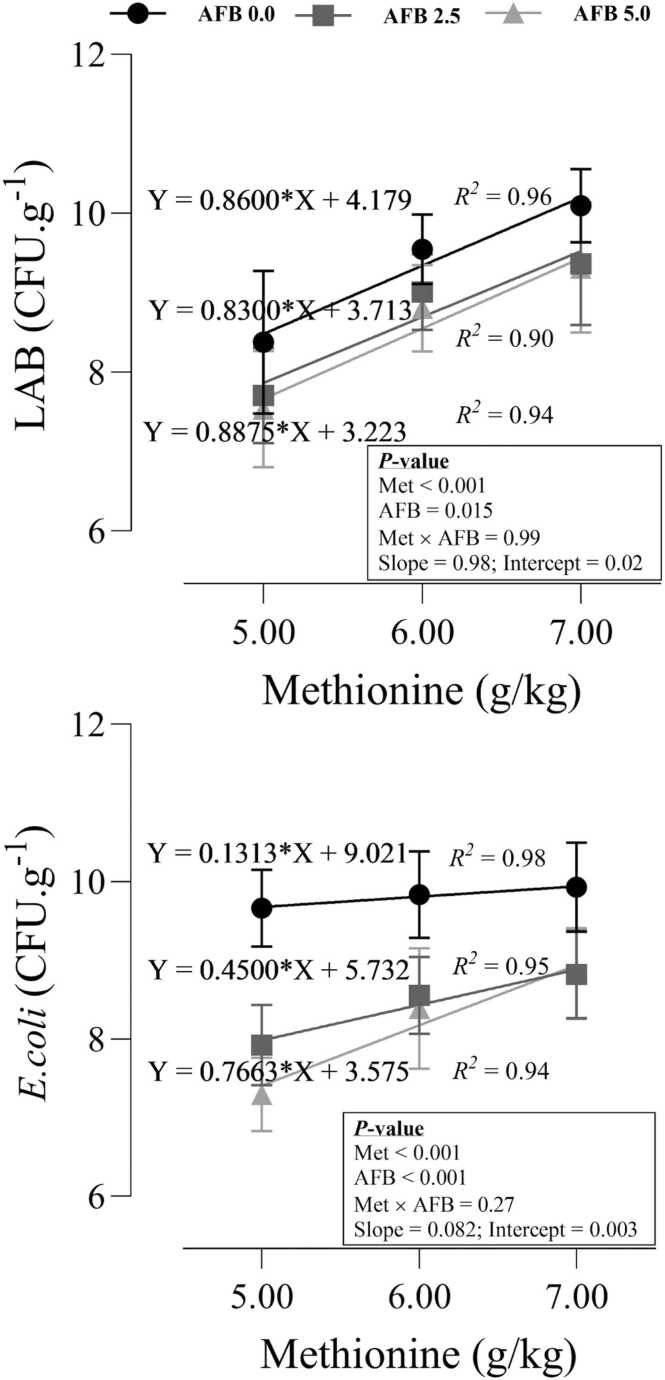
*Lactobacillus* (LAB) and E-coli populations in the intestine of quail chicks fed different levels of dietary methionine when exposed to aflatoxin B_1_. The intercept and slope of the fitted lines were evaluated statistically and probabilities of these parameters were provided.

The changes in LAB and E-coli populations in response to dietary Met and AFB_1_ were shown in Fig. 6. AFB_1_ significantly decreased (P = 0.015) the LAB colonies while increasing dietary Met increased (P < 0.001) the population of LAB bacteria. The significant intercept (P = 0.02) of the models indicated that LAB bacteria was higher in toxic-free birds than those fed AFB_1_ at all dietary levels of Met. AFB_1_ decreased (P < 0.001) the E-coli populations while the increasing dietary Met increased E-coli colonies. The significant intercept (P = 0.003) of the models indicated that E-coli colonies were higher in toxic-free birds than those fed AFB_1_ at all dietary levels of Met. However, the statistical trend of the slope of the models (P = 0.082) revealed that the efficacy of dietary Met in changing E-coli populations could be higher in birds who received AFB_1_-contaminated diets than those who received toxin-free diets.

## 4 Discussion

The present study demonstrates that aflatoxicosis induced by AFB_1_ has detrimental effects on the growth performance, health, immune system, meat quality, and intestinal microbiota of growing Japanese quail chicks. These findings align with previous research showing that mycotoxins exert more significant negative effects on growth in young poultry compared to mature birds, particularly in feed intake [17]. As a result, lower protein and methionine intake exacerbates the issue. However, increasing daily methionine intake can reduce the negative impact of AFB_1_ on BWG [10]. This highlights methionine's potential as a dietary intervention strategy against AFB_1_ in poultry.

Several methods, such as chemical, physical, and biological methods, have been employed to counteract the adverse effects of AFB_1_ in poultry. The biological method stands out as a promising approach to combat AFB_1_ toxicity, while avoiding any adverse effects on birds. For example, high doses of tryptophan have been shown to offset the negative effects of AFB_1_ on quail performance, meat quality, immunity, and microbial communities [4]. Similarly, the current study found that high concentrations of Met improved both quantitative and qualitative responses.

Although the exact mechanisms by which Met acts against aflatoxicosis are not explicitly mentioned, several potential explanations exist, including its role in protein synthesis, antioxidant defence, and immune function. Methionine may also act as an aflatoxin binder, mitigating AFB_1_ toxicity [18] and indirectly enhancing intestinal immunity and antioxidant protection [19]. In this study, there was no interaction effect between Met and AFB_1_ on FI and growth rate, but increasing dietary Met improved feed efficiency in a dose-dependent manner when challenged with AFB_1_. This suggests that methionine might regulate overall performance under aflatoxicosis via multiple cellular mechanisms, such as improving oxidative balance.

This hypothesis is supported by the observed decrease in meat MDA production in birds fed AFB_1_-contaminated diets with high dietary Met levels. As a byproduct of cellular oxidative reactions, MDA indicates severe oxidative damage. Methionine, as a precursor of glutathione (GSH), may enhance glutathione peroxidase (GPx) production, thus regulating oxidative stress by boosting the body's antioxidant system [20]. In the present study, the positive effect of Met on drip loss was negligible in the toxin-free group but significantly reduced drip loss when dietary methionine was increased in AFB_1_-contaminated diets. This suggests that the improvement in the antioxidant system was associated with cellular membrane integrity and decreasing drip loss caused by the protective role of Met either as an endogenous antioxidant involved in redox balance maintenance and stress adaptation or as an important precursor of GSH [21]. These beneficial effects of Met on meat quality and redox balance were associated with increasing meat pH, which is considered more favorable for muscle-to-meat conversion compared to acidic conditions, as a more alkaline environment helps to improve the texture and quality of meat. In acidic conditions, the muscle proteins can become denatured and aggregated, resulting in a tougher and drier texture in the meat. Additionally, an acidic environment can lead to increased water loss during cooking, which further contributes to a tougher texture and reduced flavour [22].

The present results showed that AFB_1_ increased the concentration of the studied hepatic enzymes in the blood, while the use of a high dose of Met significantly decreased those concentrations. Bagherzadeh-Kasmani, Omidikia [23] and Khanipour, Mehri [4] reported that hepatic damage caused by AFB_1_ could increase the concentration of the ALP, LDH, AST, and ALT as the blood markers in quails. We hypothesized that the negative effects of AFB_1_ on the liver as a target tissue could be ameliorated through two pathways. First, since Met acts as a precursor to glutathione, it can stimulate the body's antioxidant defence mechanism and reduce the activity of P450 in the liver, a primary location for AFB_1_ bioactivation into exo-8-9 epoxide (AFBO), a highly cancerous compound produced as a byproduct of AFB_1_ metabolism [24]. Second, dietary Met, as a precursor to glutathione, may take part in phase 2 metabolism of AFB_1_ by binding with it and promoting its excretion process [25]. As stated in the present study, a high dose of Met successfully ameliorated the negative status of the liver by decreasing the concentration of the hepatic enzymes in blood, which was in line with Khanipour, Mehri [4].

In the present study, AFB_1_ led to a decrease in glucose, TG, and TP concentrations in blood, which was in line with Khanipour, Mehri [4], Barati, Chamani [26], and Bagherzadeh-Kasmani and Mehri [27]. However, increasing dietary Met increased the blood concentrations of those nutrients, some have suggested that AFB_1_-DNA adducts can inhibit transcription or translation, and AFB_1_-lysine adducts can result in protein degradation or excretion [24], In addition, AFB_1_ can also reduce the absorption of nutrients in birds, including glucose and protein. This can lead to a decrease in the levels of these nutrients in the blood [28].

In the present study, AFB_1_ substantially impaired the humoral immunity of quail chicks, possibly through suppression of the B-cell to produce the antibodies [29], while increasing the levels of Met in their diets efficiently enhanced this immunity. The strengthening of immunity by Met could likely be associated with its antioxidant properties, as the accumulation of reactive oxygen species (ROS) in the cell by AFB_1_ is a major cause of suppression of humoral immunity [30]. Another impact of AFB_1_ on humoral immunity is the modulation of cytokine production in birds. Cytokines are signalling molecules that play a crucial role in the immune response. Modulation of cytokine expression can lead to changes in the immune response, including the humoral immune response [29].

AFB_1_ significantly reduced the number of LAB in the intestine while dietary Met reversed this negative effect. Some suggest that lactic acid bacteria could bind AFB_1_ in the intestine as a toxin binder and mitigate the adverse effects of AFB_1_ [31], leading to decrease LAB colonies in the intestine. Gut microbiota plays a critical role in maintaining intestinal health in quails by aiding digestion, enhancing immune responses, and protecting against pathogens [32]. The microbiota in the gastrointestinal tract (GIT) of growing quails has demonstrated its importance for the host's health, positively impacting the immune system, GIT physiology, and productivity [27], [33]. AFB_1_ can decrease the levels of lactic acid bacteria in the bird intestine by inhibiting their growth, disrupting the balance of gut microbiota, impairing their detoxification abilities, and increasing susceptibility to AFB_1_ toxicity [34]. The disruption of the balance of gut microbiota, brought by AFB_1_, could have led to a simultaneous decrease in E-coli populations. Meanwhile, the protective properties of dietary Met may aid in the improvement of the intestinal environment by fostering the growth and proliferation of both *Lactobacillus* and E-coli populations. Dysbiosis, or an imbalance in gut microbial populations, can impair these functions and make birds more susceptible to diseases and toxins. Unbalanced microbiota can induce inflammation, leaky gut, or other gut-related disorders [35].

## 5 Conclusion

Dietary Met supplementation demonstrated linear improvements in FI and BWG in quail chicks, independent of AFB_1_ levels. The findings indicate that increasing dietary Met levels enhances growth performance, with the most significant positive effects observed in quail chicks exposed to higher concentrations of AFB_1_ (5.0 g/kg). Dietary supplementation of methionine demonstrated beneficial effects in mitigating the negative impacts of AFB_1_ on the liver enzymes, blood biochemical profiles, and overall health of the studied birds. Met supplementation enhances humoral immunity, meat quality, and intestinal microbiota balance in AFB_1_-contaminated diets. The current study has demonstrated that increasing the dietary Met level beyond the nutritional requirements shows promise in alleviating the adverse effects of aflatoxicosis in quail chicks. However, further research is required to fully comprehend the mechanisms responsible for these protective effects and to devise practical approaches for preventing toxicity in quail chicks exposed to aflatoxin-B_1_.

## CRediT authorship contribution statement

**Farzad Bagherzadeh-Kasmani:** Writing – review & editing, Writing – original draft, Validation, Methodology, Conceptualization. **Mahmoud Ghazaghi:** Writing – review & editing, Writing – original draft, Resources, Methodology, Data curation. **Mehran Mehri:** Writing – review & editing, Writing – original draft, Supervision, Methodology, Funding acquisition, Formal analysis, Conceptualization. **Mohammad Rokouei:** Writing – review & editing, Writing – original draft, Visualization, Validation, Formal analysis, Conceptualization. **Adel Ghorbani:** Writing – review & editing, Writing – original draft, Investigation.

## Ethics approval

This experimental protocol has been approved by both the animal ethics committee at the University of Zabol and the Iranian Council of Animal Care. The study adhered to the ARRIVE guidelines and the NIH Guidelines for Animal Care and Use [8].

## Funding statement

The authors would like to express their gratitude to the University of Zabol for providing the necessary experimental facilities and financial support (grant numbers UOZ-GR-2780).

## Author statement

All authors have consented to participate in the research and have agreed to the publication of this manuscript in Toxicology Reports.

## Declaration of Competing Interest

The authors declare the following financial interests/personal relationships which may be considered as potential competing interests: MEHRAN MEHRI reports financial support was provided by University of Zabol. MEHRAN MEHRI reports a relationship with University of Zabol that includes: employment. If there are other authors, they declare that they have no known competing financial interests or personal relationships that could have appeared to influence the work reported in this paper.

## Data Availability

Data will be made available on request.
